# MSK ultrasound imaging–assisted clinical examination

**DOI:** 10.1177/1742271X241280911

**Published:** 2024-09-20

**Authors:** Nikos Malliaropoulos, Stavros Daoukas

**Affiliations:** 1 Centre for Sports and Exercise Medicine, William Harvey Research Institute, Queen Mary University of London, London, UK; 2 Sports Clinic, Rheumatology Department, Mile End Hospital, Barts Health NHS Trust, London, UK; 3 Sports and Exercise Medicine Clinic, Thessaloniki, Greece; 4 European College of Sports and Exercise Physicians (ECOSEP), Thessaloniki, Greece; 5 Institute of Health and Social Care, School of Allied and Community Health, London South Bank University (LSBU), London, UK; 6 Radiology Department, Queen’s Hospital NHS Trust, Barking, Havering and Redbridge University Hospital (BHRUT), Romford, UK; 7 Greek Athletic Therapy Association (GATA), Thessaloniki, Greece

**Keywords:** Musculoskeletal, ultrasound imaging, medical education, point-of-care-ultrasound

## Abstract

Musculoskeletal disorders are a significant global health concern, affecting over 1.71 billion individuals worldwide, with a considerable impact on quality of life and economic burden due to healthcare costs and productivity losses. In the United Kingdom, approximately one-third of the population suffers from musculoskeletal disorders, underscoring the need for effective diagnostic and management strategies. Musculoskeletal ultrasound imaging emerges as a preferred diagnostic modality, offering a balance between technical capabilities and cost-effectiveness, owing to its non-invasive nature, portability and lack of radiation exposure. However, the operator-dependent nature of musculoskeletal ultrasound imaging necessitates specialised training for medical and healthcare professionals. The integration of musculoskeletal ultrasound imaging into traditional clinical examinations, known as ultrasound imaging–assisted clinical examination (UIACE), enhances traditional diagnostic processes by providing immediate visual feedback, facilitating a more accurate and comprehensive assessment of musculoskeletal conditions. This approach not only refines diagnosis in cases with ambiguous symptoms or overlapping signs but also significantly improves patient reassurance and management strategies. In addition, incorporating musculoskeletal ultrasound imaging into medical education through ultrasound imaging–assisted clinical examination offers students a dynamic, interactive learning experience, fostering a deeper understanding of clinical anatomy and examination skills. By advocating for its systematic inclusion in the undergraduate medical curriculum, the study highlights the potential to enhance the competence and confidence of future professionals in utilising ultrasound imaging, ultimately improving patient outcomes in musculoskeletal care.

## Introduction

Musculoskeletal disorders (MDs) impose a significant burden on global health systems, affecting over 1.71 billion individuals worldwide with a range of conditions that drastically impair quality of life and independence.^1^ In the United Kingdom, MDs represent a significant health concern, impacting a substantial segment of the population. It is estimated that around one third of the population suffer from a musculoskeletal condition.^2^ The prevalence of MDs in the United Kingdom not only highlights a significant public health issue but also reflects broader impacts on healthcare services and the economy. While the substantial economic burden of MDs is partly due to direct healthcare costs and productivity losses, the escalating expense of healthcare is amplified by the reliance on advanced diagnostic imaging modalities.^3^

## Musculoskeletal ultrasound imaging

Among the array of diagnostic imaging modalities, musculoskeletal ultrasound imaging (MUSI) has emerged as a popular and viable option not only due to its non-invasive nature, portability, dynamic imaging potential and lack of exposure to radiation but also owing to its balance of technical capabilities and cost-effectiveness.^4^

The broad application of MUSI spans various domains of medicine and allied health professions (AHPs), including rheumatology, orthopaedics, sports medicine, radiography, physiotherapy and podiatry, among others. Its capacity for detailed assessment of tendons, ligaments, muscles and nerves makes it an invaluable tool in diagnosing a wide array of conditions, from tendinosis and muscle tears to arthropathies and nerve entrapments.^4^ The advent of portable ultrasound devices has expanded the utility of MUSI beyond traditional settings, enabling point-of-care ultrasound (PoCUS) diagnostics and interventions in various environments.^5^

Despite these advantages, the reliance on MUSI necessitates a high level of skill and expertise for accurate image acquisition and interpretation. The operator-dependent nature of MUSI underscores the importance of specialised training and continuous education for healthcare professionals to maintain proficiency.^6^

MUSI use is increasingly recognised among medical and AHPs, as highlighted by emerging research.^7,8^ The increasing preference for ultrasound over more costly and invasive diagnostic modalities not only reflects a shift towards more efficient and patient-centred care but also underscores the need for a more systematic approach to its implementation across all medical and healthcare disciplines. Yet, the adoption and application of MUSI across the spectrum of the medical and AHP domain, particularly by those specialising solely in the management of MDs, remain underexplored.

## Ultrasound imaging–assisted clinical examination

Clinical examination remains a cornerstone of initial clinical assessment, yet its limitations are recognised in cases where symptomatology is ambiguous or when conditions present with overlapping signs. MUSI has emerged as an adjunct, offering the capability to refine diagnosis through ‘in-room’ scanning, that extends the traditional physical examination,^9,10^ particularly in conditions where clinical symptoms may be non-specific or mimic other pathologies. The integration of MUSI into clinical practice has been shown to enhance patient reassurance through immediate visual feedback and play a crucial role in tailoring management strategies, thus improving patient outcomes.^9,11^

MUSI can revolutionise clinical examination and education for medical students by offering a dynamic and interactive approach to learning. The ultrasound imaging–assisted clinical examination (UIACE) represents a significant advancement in medical education, providing students with invaluable opportunities to enhance their understanding and skills.

One of the key benefits of ultrasound imaging in medical education is its ability to provide real-time visualisation of anatomical structures. Through UIACE, students can gain a deeper understanding of clinical anatomy by directly visualising internal organs, tissues and musculoskeletal structures. This hands-on experience allows students to correlate theoretical knowledge with practical application, thereby reinforcing their understanding of anatomical relationships and variations.^12^

In addition, ultrasound imaging serves as a powerful tool for assisting students’ education in their clinical examination skills. By incorporating ultrasound into clinical examination sessions, students can visualise the internal structures being examined, enabling them to identify abnormalities, assess function and make more accurate diagnoses. This direct feedback enhances students’ clinical reasoning abilities and fosters a more comprehensive approach to patient care. In addition to improving clinical examination skills, ultrasound imaging enhances students’ knowledge of both normal and abnormal anatomy and the imaging presentation of certain injuries. By observing the characteristic sonographic features of various pathologies, students develop a deeper understanding of disease processes and diagnostic criteria. This exposure to real-life clinical cases helps bridge the gap between theoretical learning and clinical practice, preparing students for the complexities of patient care.

Ultrasound imaging also facilitates the development of new skills among medical students. Through hands-on experience with ultrasound equipment, students learn how to acquire and interpret images, improve physical examination and evaluation skills, manage patient outcomes, understand ultrasound-guided procedures and integrate findings into patient care plans. These practical skills are invaluable for future medical practice, empowering students to provide high-quality care and contribute to advancements in healthcare.

## Conclusion

Overall, ultrasound imaging plays a crucial role in enhancing clinical examination and education for medical students. By providing real-time visualisation, assisting in clinical examinations, improving knowledge of injuries and fostering the development of new skills, ultrasound-based education prepares students to excel in their future careers as healthcare professionals.

We hope this commentary acts as a call to action for the sport and exercise medicine research community to further explore the integration and impact of ultrasound imaging within the spiral curriculum of undergraduate medical education. By investigating how this curriculum model can enhance the learning and application of MUSI across various stages of education, we can uncover strategies to build competence and confidence in its use among medical students.
